# The effect of water-based plyometric training on vertical stiffness and athletic performance

**DOI:** 10.1371/journal.pone.0208439

**Published:** 2018-12-06

**Authors:** Daniel Sporri, Massimiliano Ditroilo, Elizabeth C. Pickering Rodriguez, Richard J. Johnston, William B. Sheehan, Mark L. Watsford

**Affiliations:** 1 Human Performance Research Centre, Faculty of Health, University of Technology Sydney, Australia; 2 School of Public Health, Physiotherapy and Sports Science, University College Dublin, Dublin, Ireland; Sao Paulo State University - UNESP, BRAZIL

## Abstract

Since higher vertical stiffness is related to superior athletic performance, training has traditionally been aimed at augmenting this variable to enhance neuromuscular output. However, research has linked elevated stiffness with increased injury risk, therefore, this study examined the effect of a novel training intervention on vertical stiffness and athletic performance. Vertical stiffness, jump performance and athletic performance were assessed in two randomly allocated groups, prior to, and following, an eight-week period. One group was exposed to a training intervention involving aqua-based plyometrics (*n* = 11) over the 8 weeks while the other acted as a control group (*n* = 9). The training intervention involved hopping, jumping and bounding in water at a depth of 1.2m whilst control participants performed their normal training. There were no significant changes in vertical stiffness in either group. Countermovement jump height and peak power significantly increased within the aqua plyometric group (*p* < 0.05). Athletic performance markers improved in the aqua plyometric group as measured using an agility and a 5-bound test exhibiting superior values at the post-test (*p* < 0.05). The results suggest that an aqua plyometric training program can enhance athletic performance without elevating stiffness. The increase in athletic performance is likely due to a reduction in ground reaction forces created by the buoyancy of the water, causing a shorter amortization phase and a more rapid application of concentric force. The findings from this study can inform exercise professionals and medical staff regarding the ability to enhance neuromuscular performance without elevating vertical stiffness. This has implications for improving athletic performance while concurrently minimising injury risk.

## Introduction

Success for individual and team sport athletes is determined by many characteristics, with one important aspect being the stiffness of the muscle-tendon unit. Exercise interventions aimed at increasing stiffness for the purpose of improving athletic performance have primarily focused on different types of resistance training. Literary findings have revealed that isometric training can increase muscle-tendon stiffness in healthy males [1, 2], while eccentric and isotonic strength training can enhance tendon-aponeurosis stiffness [3, 4]. Further, relatively higher levels of stiffness have been associated with enhanced athletic performance during fast stretch-shorten cycle (SSC) activities [5, 6]. Despite growing evidence of a positive relationship between stiffness levels and muscular performance, interestingly, stiffness may also influence injury risk. Reduced stiffness may have implications for soft tissue injury due to excessive joint motion, while higher values of stiffness may be associated with greater risk of bone injury due to increased peak forces [7]. This suggests that there may be an optimal range for stiffness that is advantageous for improved athletic performance whilst minimising the risk of injury [7].

Given the potential link between elevated stiffness and injury, it appears that optimisation in stiffness levels may indeed act as an injury prevention strategy. A recent study reported that the percentage difference between legs in lower-limb stiffness was related to in-season injuries in professional Australian footballers [8]. Therefore, the ability to balance stiffness levels between limbs may be of equal importance to exercise practitioners since many athletes display intra-varying stiffness results. To date, the main intervention strategy for reducing stiffness has been flexibility training [9, 10], however it is well established that such training can be detrimental to athletic performance, particularly sprint performance due to a potential reduction in rate of force development (RFD) [11–13]. It is therefore important to develop strategies that optimise performance while minimising risk of injury.

Similar to flexibility training, there is evidence suggesting that manipulating ground surfaces can consequently modify lower limb stiffness [14–16]. Despite this, there is limited evidence demonstrating chronic reductions in lower-body stiffness from such practises. The neuromuscular properties associated with changes in stiffness seem to be limited by the mechanisms of these training interventions on different ground surfaces. Interestingly, the modification of the training environment has been the focus of recent reports investigating aqua-based exercise programs. These studies revealed that the changes in ground reaction forces led to improvements in athletic performance [15, 17]. Specifically, the buoyance of the water provides a mechanism which reduces peak vertical ground reaction forces upon landing, allowing for a shorter amortization phase, with fluid drag creating resistance during propulsive motions [17–19]. As a result, aqua plyometric training programs have significantly increased athletic performance outputs such as muscle power, vertical jump performance, isokinetic torque and sprint performance whilst concurrently reducing muscle soreness [14–16]. Despite improvement in performance variables, stiffness was not concurrently measured in these studies. Examination of stiffness variables, alongside this form of training, may be warranted as there is the potential for mechanical and myogenic adaptions from a buoyant environment exists and these adaptations may impact upon stiffness. Accordingly, the aim of this study was to investigate modifications in stiffness levels following the use of a dynamic training program in a water environment. It was hypothesised that such a training intervention would improve athletic performance while minimising alterations in stiffness due to the reduction in ground reaction forces.

## Methods

### Experimental approach to the problem

Males who were actively engaged in team sports were recruited and assessed for vertical stiffness (K_vert_) and athletic performance markers including strength, power, agility and acceleration. Participants were randomly allocated to either an aqua plyometric experimental (APG) or control group (CG). The participants in the experimental group completed a novel eight-week plyometric training program, while the control group refrained from additional exercise outside their normal daily activities. A training period of eight-weeks was deemed sufficient to induce power related adaptations as previous training studies spanning 3–12 weeks have demonstrated improvements in power output in elite athletes [20]. Following the training period, all participants were re-tested, and the data was analysed using standard repeated measures statistical procedures. The use of a broad range of neuromuscular assessments in conjunction with a novel training program has implications for the prescription of exercise across a number of populations.

### Subjects

Twenty-one, sub-elite, active male team sport participants (22.2 ± 2.5 years, 77.7 ± 8.0 kg and 1.80 ± 0.08 m) volunteered to participate in this study with written informed consent. The subjects participated in team sports such as rugby union, rugby league, Australian Rules Football, basketball or soccer 3 times per week for a mean of 3.5 hours per week of training and match-play and had 4 ± 2 years of experience. Subjects had no history of formal plyometric training apart from that incidentally performed as part of their training and match-play were required to be free from injury during the six months prior to participating and be aged between 18–30 years. The study was granted ethical approval by the Human Research Ethics Committee of the University of Technology Sydney. Before participation, all subjects were informed of the benefits and risks of the investigation prior to signing an institutionally approved informed consent document.

### Procedures

#### Vertical stiffness assessment (K_vert_)

The unilateral hop test was used to quantify K_vert_ due to reportedly excellent reliability and validity [8, 21]. Participants completed a five-minute aerobic warm-up, with no static stretching prescribed prior to testing. They were required to hop unilaterally at a rate of 2.2Hz [21] on both their dominant (DOM) and non-dominant (NON-DOM) leg previously specified by the participant [8]. Participants were required to complete the test bare-footed to negate force absorption from shoes, with their hands on their hips to prevent gaining momentum from upper-body movements. A rest period of 60 seconds was permitted between inter-limb trials. The test was performed on a 900mm x 600mm 1-dimensional force plate (Onspot, Wollongong, NSW) with sampling frequency of 1000Hz. The unilateral hopping test has reported high reliability with a TEM of 4.15% and intra-class correlation of 0.80 [22]. A 16-bit analog/digital data converter (National Instruments, Austin, TX, USA) along with customised data acquisition software was used along with customised analysis software (Microsoft Visual C++, Redmond, WA, USA). For each trial, force-time data was collected for 5–7 seconds of hopping, resulting in approximately 12–15 hops per trial. Force data was evaluated immediately following each trial to ensure the hops fell inside ±2% of the prescribed frequency. Three consecutive hops within each trial where the ground reaction force was deemed stable via a visual inspection were used for subsequent analysis with K_vert_ (N·m^-1^·kg^-1^) determined as the ratio of maximum ground reaction force and maximum vertical displacement of the centre of mass [7]. Centre of mass displacement was calculated by double integration of the force-time curve [23]. The variables quantified from the unilateral hop test included dominant limb K_vert_ (DOM K_vert_), non-dominant K_vert_ (NON-DOM K_vert_), the average of these two values (K_vert_ MEAN) and bilateral K_vert_ percentage asymmetry (K_vert_ ASYMM), calculated as:

$${\text{K}}_{\text{vert}}\text{ASYMM}=\left[\left({\text{highest}\;\text{K}}_{\text{vert}}-{\text{lowest}\;\text{K}}_{\text{vert}}\right)/{\text{lowest}\;\text{K}}_{\text{vert}}\right]\;\times 100$$

#### Jump performance

A countermovement jump (CMJ) was included as a measure of athletic performance due to its relationship with peak lower-body power output while a squat jump (SJ) was performed due to its representation of lower-body concentric power development [24–26]. Both jumps provide inside into the restitution of elastic energy [25]. For both types of jumps, after two practice attempts, participants were instructed to perform two maximal attempts with hands on their hips, with a 90 seconds recovery period between trials. The jumps exhibiting the highest vertical displacement were used for analysis. Participants were given no specific instructions or restrictions regarding the amount of eccentric lowering of the hips during jumping but were instructed to jump as high as possible. Jump height was recorded, along with peak rate of force development (RFD), peak velocity and peak power which were determined from the concentric part of the curve prior to take-off.

A drop jump (DJ) was assessed to determine the reactive strength of the lower-body [27]. All participants performed a DJ off a 0.5m box and were instructed to keep their hands on their hips, land bilaterally, minimise ground contact time, and jump for maximum height. As with the CMJ and SJ, familiarisation trials were permitted before two maximal jumps were recorded with the best trial used for analysis. A recovery period of two minutes was implemented between each DJ. The outcome for the DJ was recorded as the reactive strength index (RSI) [28] calculated as:

$$\text{RSI}\;=\text{Jump}\;\text{Height}\;\left(\text{cm}\right)\cdot \text{Contact}\;\text{time}\;{\left(\text{msec}\right)}^{\text{-1}}\times \;\text{100}$$

#### Athletic performance

An indoor 10m sprint test was used to measure velocity, horizontal power and maximum acceleration. Both the 5m and 10m splits were recorded during the same sprint trial using timing lights (Swift Performance, Queensland, Australia). Participants placed one foot at the start line and were instructed to run in the shortest time possible to the 10m markers, ensuring maximum acceleration until at least the 10m mark. All participants performed one practice trial before completing two maximal sprint efforts separated by two minutes of passive recovery. The fastest 10m trial was used for further analysis.

The 5-0-5 test assesses agility capabilities through a deceleration followed by maximal acceleration [6]. The agility course required participants to sprint for 5m following an untimed 10m commencement run, stop as quickly as possible, turn 180° on their self-selected foot, and then accelerate maximally for 5m in the returning direction. A timing gate was placed at the start/finish line to quantify the time to complete the course, with all participants completing two maximal efforts, separated by a two-minute recovery period. The best result for each participant was recorded for further analysis.

The 5-bound test required participants to complete five consecutive steps, aiming for maximum horizontal displacement [29, 30]. Participants were permitted two familiarisation trials before completing two maximal tests interspersed with two minutes of recovery between each bout. A 20m measuring tape lay next to the bounding track with close invigilation of the front foot landing on the fifth bound used to determine the horizontal distance achieved over five bounds. The trial that achieved the greatest horizontal displacement was used for further analysis.

#### Training protocol

Following the pre-testing and random group allocation, the participants in the APG commenced the aqua-based plyometric training program within 72 hours. The program was eight weeks in duration and consisted of three training sessions per week, separated by at least 24 hours. The training program was completed in an indoor 25m pool, with a constant depth of 1.2m and a water temperature of 29°±2°. Exercises that utilise the stretch shorten cycle to develop power during the movement were incorporated into the program and included unilateral and bilateral movements such as jumping, hopping and bounding. The program progressed participants from two sets of eight repetitions to three sets of fifteen repetitions per exercise with session totals ranging from 56 to 138 ground contacts. All sessions began with a 5-minute progressive dynamic warm-up. Participants were instructed to perform each exercise with maximal effort and to limit ground contact time. 60–120 seconds of passive recovery was prescribed between each exercise to allow adequate recovery. To ensure no propulsion from the upper-body during any of the exercises, participants were instructed to keep their hands on hips. The CG were not prescribed any formal training, rather they were instructed to complete their regular exercise regimes throughout the eight-week period.

### Statistical analyses

Standard statistical procedures for the determination of means and standard deviations were applied with pre- and post-test means compared for each of the dependent variables. All data was processed using statistical software (Statsoft, Inc. 2014. Version 12, Tulsa, OK, USA) and all relevant data are contained within this paper. All sets of data were assessed for normality using Shapiro-Wilkes test while Levene’s test for homogeneity of variance revealed homoscedasticity across results. Between-group differences at the pre-test were assessed using independent samples t-test. A 2 (groups) x 2 (Pre-Post condition) ANOVA with repeated measures on the last factor was employed to identify any differences following the training program. If a significant F value was present, Tukey’s post hoc tests were used to establish where the differences lay. A significant difference was established when an alpha level of *p* ≤ 0.05 was recorded. To assist with the quantification of trends in the data, quasi-significant differences were defined with an alpha level between 0.05 and 0.10 [31]. Effect sizes for the repeated measures ANOVA were calculated using partial η^2^ with magnitudes considered to be small (< 0.06), moderate (0.06–0.14) and large (> 0.14), while Cohen’s *d* was used to quantify the effect size difference within groups, with small (< 0.49), moderate (0.5–0.79) and large (> 0.80) magnitudes considered [32].

## Results

### Vertical stiffness (K_vert_)

During the training program, one participant dropped out from the CG for reasons unrelated to the training. This resulted in 11 participants in the APG and 9 in the CG, each of whom completed all testing and training sessions. The K_vert_ results are summarised in Table 1. Dominant limb K_vert_ and K_vert_ MEAN differed between groups at the pre-test (p < 0.05). No significant changes were evident for any of the variables following the training period, however, a quasi-significant interaction with a large effect size between factors was identified with an 8.1% reduction in DOM K_vert_ following the training (F = 4.38, *p* = 0.05, partial η^2^ = 0.20). Whilst not significant, there was a tendency for a moderate 6.9% decrease in K_vert_ ASYMM (6.9%, *d* = 0.53) between the pre- and post-test in the APG. Additionally, a small effect was demonstrated in each group with a 4.5% decrease in K_vert_ MEAN (*d* = 0.19) recorded at the post-test for the APG contrast with a 5% increase in the CG (*d* = 0.24).

**Table 1:** Mean ± standard deviation for vertical stiffness and associated variables.

	Test	Control group (n = 9)	Aqua Plyometric Group (n = 11)
Dominant limb K_vert_ (N·m^-1^·kg^-1^) **	Pre	148 ± 24.6	175 ± 24.8 †
	Post	154 ± 29.1	161 ± 16.3
Non-Dominant limb K_vert_ (N·m^-1^·kg^-1^)	Pre	146 ± 25.1	164 ± 25.8
	Post	152 ± 28.5	163 ± 17.1
K_vert_ Asymmetry (%)	Pre	8.94 ± 8.21	12.0 ± 9.55
	Post	7.87 ± 5.82	5.08 ± 3.57
K_vert_ MEAN (N·m^-1^·kg^-1^)	Pre	147 ± 23.7	169 ± 22.4 †
	Post	153 ± 28.0	162 ± 15.9
Peak Force mean (N)	Pre	2050 ± 314	1991 ± 358
	Post	2178 ± 406	2039 ± 240
COM Displacement mean (m)	Pre	0.17 ± 0.03	0.16 ± 0.02
	Post	0.18 ± 0.03	0.17 ± 0.01

^†^ Significantly different to control group pre-test;

** Quasi-significant condition x pre-post interaction (0.05 ≤ *p* ≤ 0.10, large ES); COM, Centre of mass; K_vert_, Vertical stiffness; Note: "MEAN" values refer to the average of dominant and non-dominant limbs.

### Jump performance

The results for the three types of jumps are reported in Table 2. Several variables recorded between-group differences at the pre-test (CMJ height, CMJ power, SJ power, all p < 0.05). The CMJ exhibited a significant interaction between factors for jump height (F = 5.02, *p* < 0.05, partial η^2^ = 0.22) and peak power (F = 5.61, *p* < 0.05, partial η^2^ = 0.24). The peak velocity showed a quasi-significant interaction (F = 3.98, *p* = 0.06, partial η^2^ = 0.18). The SJ showed a significant interaction for peak velocity (F = 8.41, *p* < 0.01, partial η^2^ = 0.32), with post-hoc tests revealing a large effect size between pre- and post-training in the APG only (+15.2%, *p* < 0.05, *d* = 0.98). Additionally, a significantly large effect pre- and post-intervention was revealed for peak power between groups (F = 8.48, *p* < 0.01) with only the APG group demonstrating a large effect following training (+17.5%, *p* < 0.05, *d* = 0.96). A quasi-significant large interaction effect was recorded in the DJ-RSI variable (F = 4.17, *p* = 0.05, partial η^2^ = 0.19).

**Table 2:** Mean ± standard deviation for counter movement jump, squat jump and drop jump variables.

	Test	Control Group (n = 9)	Aqua Plyometric Group (n = 11)
CMJ Height (cm)*	Pre	38.6 ± 5.20	33.6 ± 3.90 †
	Post	36.4 ± 6.30	35.9 ± 5.50
CMJ Peak velocity (m.s^-1^)**	Pre	3.13 ± 0.60	2.75 ± 0.22
	Post	2.80 ± 0.42	2.94 ± 0.58
CMJ Peak Power (W)*	Pre	484 ± 103.5	378 ± 31.6 †
	Post	442 ± 119	424 ± 76.1
SJ Height (cm)	Pre	31.0 ± 8.70	30.4 ± 3.60
	Post	32.5 ± 5.80	32.1 ± 5.10
SJ Peak velocity (m.s^-1^)*	Pre	2.56 ± 0.40	2.23 ± 0.53
	Post	2.68 ± 0.21	2.63 ± 0.29 #
SJ Peak Power (W)***	Pre	419 ± 90.3	308 ± 82.5 †
	Post	444 ± 78.7	373 ± 54.0 #
DJ Reactive strength index (cm.msec^-1^)**	Pre	13.3 ± 4.00	9.90 ± 4.00
	Post	12.2 ± 4.50	11.4 ± 4.30

^†^ Significantly different to control group pre-test;

*Significant condition x pre-post interaction (p<0.05, large ES);

**Quasi-significant condition x pre-post interaction (0.05≤p≤0.1, large ES);

***Significant pre-post differences (p<0.01, large ES);

^#^ Significantly different from Pre-test (p<0.05); CMJ, Countermovement jump; SJ, Squat jump; DJ, Drop Jump.

### Athletic performance

The results of the performance tests are summarised in Table 3. A between-group difference was noted at the pre-test for the measure of agility (p < 0.05). A quasi-significant interaction with a large effect was evident for the 10m sprint test (F = 3.55, *p* = 0.08, partial η^2^ = 0.16), whereas both the agility test and the 5-bound test exhibited a significant pre- and post-training difference (F = 8.39, *p* < 0.01, partial η^2^ = 0.32; F = 14.48, *p* < 0.01, partial η^2^ = 0.45, respectively). Additionally, an interaction difference was revealed pre- and post-intervention (F = 12.84, *p* < 0.01, partial η^2^ = 0.42; F = 7.47, *p* < 0.05, partial η^2^ = 0.29 respectively). Post-hoc tests revealed significant differences and large effects between pre- and post-training for agility (-10.2%, *p* < 0.01, *d* = 1.93) and 5-bound test (+8.1%, *p* < 0.01, *d* = 1.17) in the APG only.

**Table 3:** Mean ± standard deviation for physical performance tests.

	Test	Control Group (n = 9)	Aqua Plyometric Group (n = 11)
5 m sprint (s)	Pre	1.23 ± 0.34	1.13 ± 0.14
	Post	1.21 ± 0.14	1.16 ± 0.06
10 m sprint (s)**	Pre	1.92 ± 0.10	1.94 ± 0.06
	Post	1.99 ± 0.17	1.93 ± 0.07
Agility test (s)*	Pre	2.54 ± 0.20	2.73 ± 0.15 †
	Post	2.57 ± 0.23	2.45 ± 0.14 #
5-Bound test (m)*	Pre	11.6 ± 1.48	11.2 ± 0.84
	Post	11.7 ± 1.52	12.2 ± 0.84 #

^†^ Significantly different to control group pre-test;

* Significant condition x pre-post interaction (p<0.05, large ES);

** Quasi-significant condition x pre-post interaction (0.05≤p≤0.1, large ES);

^#^ Significantly different from Pre-test (p<0.01)

## Discussion

This study examined the effectiveness of plyometric training in an aquatic environment with the aim of improving athletic performance while concurrently monitoring stiffness. The results revealed that aqua-based plyometric training did not affect K_vert_ MEAN, however there was a tendency for moderate and large reductions in K_vert_ ASYMM and DOM K_vert_, respectively. This highlights that aqua-based plyometrics may affect neuromuscular properties. An absence of change in K_vert_ MEAN may be a consequence of fluid dynamics associated with training in water. The buoyancy of the water reduces ground reaction force upon landing, leading to lower eccentric loading during hopping or jumping in water [18]. This creates temporal changes in muscle recruitment, due to the reduction in contact time during the amortization phase, and potentially allows for improved stretch-shorten cycle performance [15] and enhanced power output [18]. This concept may also explain why there were no changes in K_vert_ MEAN measures as the modification to vertical ground reaction forces may have mitigated any neuromuscular changes that affect K_vert_. However, over a longer period of training, greater than that of the 8-weeks in this study, continuous exposure to reduced ground reaction forces may manifest into augmented chronic adaptations. Additionally, training in an aquatic environment may enhance cross-neural activation resulting in a decrement in neuromuscular asymmetry as displayed by the moderate reduction in K_vert_ ASYMM (6.9%) in the APG. However, the large tendency for reduced DOM K_vert_, compared with an absence of change in NON-DOM K_vert_, indicates that neuromuscular modifications may only occur in the dominant limb. The apparent discrepancies between limbs warrants further investigation in order to elucidate the mechanisms causing these neuromuscular changes.

A key component of this study and the associated hypothesis was to identify a training protocol that could improve athletic performance whilst concurrently mitigating unwarranted changes in lower-limb stiffness. Following the training period, the results highlight that certain athletic performance markers improved, including jumping ability, agility and bounding performance while maintaining, and even decreasing, selected K_vert_ measures. These results confirm the hypothesis of the study and provide valuable information for practitioners. Plyometric training on land has been reported to increase stiffness and performance [6], however the findings of the current study revealed that aqua plyometrics improved performance markers without elevating K_vert_. This may be of relevance to practitioners as an improvement in performance in the absence of elevations in K_vert_ is highly desirable given the reported relationships between K_vert_ and injury [7, 33].

The improvements in jump performance, in the absence of increase in K_vert_, may be attributed to increase in strength following the training protocol. During concentric movements in water, the body of fluid provides a resistance with the size and shape of an individual, along with speed of movement, dictating that level of resistance. A significant increase in CMJ height and peak power following aqua plyometric training may be due to a number of factors including improved concentric strength [14, 15]. Additionally, the results of this study suggest that the buoyancy of the water played an important role in modifying neuromuscular recruitment as a decrease in ground reaction force, and more importantly, a reduced amortization phase, is integral to improving CMJ performance as it enhances the transfer of elastic energy within muscles during this phase [34, 35]. The magnitude of increase in CMJ height was similar to those previously reported following aqua plyometrics, where a significant increase in vertical jump height [14, 16] and isokinetic peak torque [16] were recorded. In the current study, improvements in DJ performance aligned with CMJ performance as a quasi-significant improvement was evident in the APG. While a decrease in vertical ground reaction force during aqua training would likely expedite the amortization phase, it is important to consider the type of exercise, level of intensity and landing technique as these factors influence the magnitude of vertical ground reaction force [17–19]. Interestingly, in the absence of change in K_vert_ measures, a significant increase in SJ peak velocity was identified within the APG, signifying strength and power adaptations. Since the SJ has no eccentric loading phase, the improvement in SJ performance can likely be explained using fluid dynamics as the drag force resulting from the water acts as a resistance to movement during the concentric phase. This concept was similarly recognised in previous research [14, 15]. Future research examining the aforementioned components in greater detail would elucidate the mechanisms responsible for these neuromuscular adaptations. Specifically, the use of a longer training period might invoke more substantial improvements in neuromuscular outputs, and the comparison of training responses from novice or untrained athletes would also be of interest.

The improvements in the 5-0-5 agility and 5-bound tests denote improvements in agility and horizontal power production. Additionally, there was a quasi-significant interaction for 10m sprint performance with the APG yielding superior results. A shorter amortization phase and an increase in resistance from fluid drag during concentric movements in an aquatic environment is likely to enhance neuromuscular recruitment and myogenic properties, enabling greater force production. Traditional land-based training interventions aimed at improving these athletic markers have often coincided with an increase in K_vert_ [5, 6] and potentially an elevated risk of soft-tissue injury [8, 33]. However, the displayed improvements in athletic performance following aqua-based plyometric training, without alterations in K_vert_ measures, are of interest to exercise and rehabilitation professionals.

A strength of this research was the prescription of a novel training program in an aquatic environment, however, the findings must be considered in conjunction with the noted limitations of the study design. These include the relatively short training period and the use of a control group where training load was not matched with the experimental group. Further, despite best efforts to match participants across the groups, some differences in key variables were evident at the pre-test which may affect the resultant training outcomes. While being mindful of these limitations, enhancing neuromuscular strength to improve athletic performance without increasing K_vert_ or inducing high compressive loads due to relatively stiffer ground reaction force environments are valuable findings from this study, with wide-ranging applications for athletes, sport scientists and physical therapists.

## Practical applications

This study examined the effect of aqua plyometric training on athletic performance while concurrently monitoring K_vert_ in the lower-limbs. The results revealed no alterations to K_vert_, yet the aqua plyometric program enhanced athletic performance. This information is of value for practitioners as aqua-based plyometric training can improve athletic performance without elevating the risk of soft-tissue injury associated with higher K_vert_. Additionally, the moderate trend for reduced K_vert_ ASYMM following aqua-based plyometric training program is valuable to practitioners as this training mode may be used as a method to potentially mitigate the risk of injury in athletes.
